# A bioorthogonal chemistry approach to detect the K1 polysialic acid capsule in *Escherichia coli*†

**DOI:** 10.1039/d2cb00219a

**Published:** 2022-12-22

**Authors:** Vincent Rigolot, Yannick Rossez, Christophe Biot, Cédric Lion

**Affiliations:** a Univ. Lille, CNRS, UMR 8576 – UGSF – Unité de Glycobiologie Structurale et Fonctionnelle Lille France cedric.lion@univ-lille.fr christophe.biot@univ-lille.fr yannick.rossez@univ-lille.fr

## Abstract

Most *Escherichia coli* strains associated with neonatal meningitis express the K1 capsule, a sialic acid polysaccharide that is directly related to their pathogenicity. Metabolic oligosaccharide engineering (MOE) has mostly been developed in eukaryotes, but has also been successfully applied to the study of several oligosaccharides or polysaccharides constitutive of the bacterial cell wall. However, bacterial capsules are seldom targeted despite their important role as virulence factors, and the K1 polysialic acid (PSA) antigen that shields bacteria from the immune system still remains untackled. Herein, we report a fluorescence microplate assay that allows the fast and facile detection of K1 capsules with an approach that combines MOE and bioorthogonal chemistry. We exploit the incorporation of synthetic analogues of *N*-acetylmannosamine or *N*-acetylneuraminic acid, metabolic precursors of PSA, and copper-catalysed azide–alkyne cycloaddition (CuAAC) as the click chemistry reaction to specifically label the modified K1 antigen with a fluorophore. The method was optimized, validated by capsule purification and fluorescence microscopy, and applied to the detection of whole encapsulated bacteria in a miniaturized assay. We observe that analogues of ManNAc are readily incorporated into the capsule while those of Neu5Ac are less efficiently metabolized, which provides useful information regarding the capsule biosynthetic pathways and the promiscuity of the enzymes involved. Moreover, this microplate assay is transferable to screening approaches and may provide a platform to identify novel capsule-targeted antibiotics that would circumvent resistance issues.

## Abstract 2

A metabolic oligosaccharide engineering method that enables bioorthogonal labelling of the K1 capsule expressed by pathogenic *Escherichia coli* strains associated with neonatal meningitis and sepsis.

## Graphical abstract

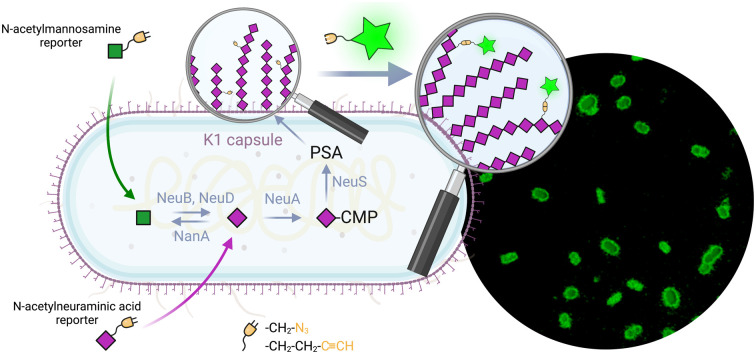

## Introduction

**Fig. 1:**
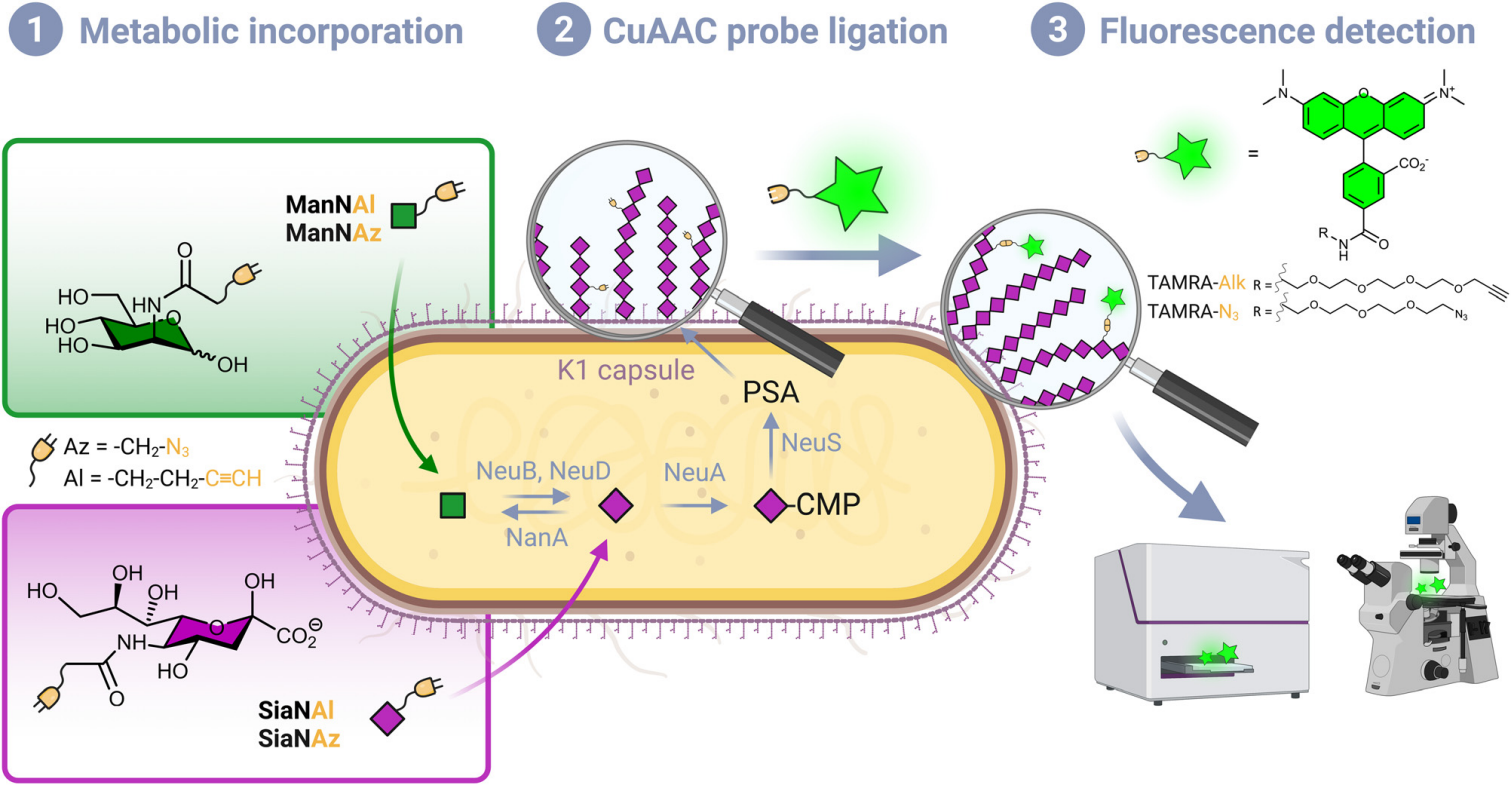
Labelling strategy workflow. (1) Chemical reporters bearing an azide or alkyne moiety are incorporated in the capsule of living *E. coli* EV36 by hi-jacking the polysialic acid metabolic pathway. After being transported from the extracellular milieu to the cytosol, ManNAc analogues enter the *de novo* biosynthesis of sialic acid (Neu5Ac), while Neu5Ac (2) the incorporated analogues are specifically labelled with a fluorescent dye *in vivo* by a CuAAC reaction. (3) The labelled bacteria are analysed with the desired technique such as fluorescence microscopy or fluorescence plate reader.

Although *Escherichia coli* is an important part of the commensal microbiota colonizing the digestive tract of humans and animals, certain strains of this species have developed virulence attributes and cause serious diseases. Among these pathogenic strains, the meningitis/sepsis-associated *E. coli* (MNEC) pathotype is an important cause of neonatal infections. Mostly transmitted from mother to infant during birth, they are known to migrate to the vascular system after mucosal colonization, then penetrate the blood–brain barrier. This generally leads to meningitis or to septicaemia (blood-poisoning), both life-threatening conditions with high mortality and morbidity rates.^1^ 80% of MNEC strains express the K1 capsule, a mucous layer of poly-α-2,8-sialic acid (PSA) that surrounds the bacterium thereby shielding its immunogenic proteins from detection by the host.^2,3^ Mimicking the human glycan structure found in the neural cell adhesion molecule (NCAM), PSA is not recognized as an external threat and allows the encapsulated bacteria to evade the immune system, and the severity of these infections is directly related to the amount of K1 antigen found at the bacterial surface.^4–7^ This capsular polysaccharide is a linear homopolymeric chain constituted of *N*-acetylneuraminic acid (Neu5Ac) monomers belonging to the sialic acid (Sia) family of carbohydrates.^8^ Its *de novo* biosynthesis requires the condensation of *N*-acetylmannosamine (ManNAc) and phosphoenolpyruvate to form Neu5Ac, which is then converted to the sugar nucleotide donor cytidine 5′-monophospho *N*-acetylneuraminic acid (CMP-Neu5Ac) and assembled into PSA in the cytosol prior to translocation to the cell surface^9^ (Fig. 1). *E. coli* serotype K1, as well as other bacteria that produce polysialic acid capsules such as *Neisseria meningitidis*, *Pasteurella haemolytica* A2 or *Moraxella nonliquefaciens*, can also scavenge free Neu5Ac from the host.^10^

Monosaccharide analogues modified with an additional chemical moiety can be used as molecular tools to engineer sialylated glycoconjugates in metabolic oligosaccharide engineering (MOE) approaches. Reutter and co-workers first pioneered MOE, with synthetic ManNAc analogues bearing an elongated *N*-acyl side chain that were successfully metabolized into cell surface sialoglycoproteins.^11^ Modern labelling methodologies combine MOE and click chemistry or bioorthogonal chemistry, an ensemble of reactions originally pioneered by the groups of Sharpless, Meldal and Bertozzi, who were recently awarded the Nobel prize for this achievement,^12–14^ and further developed by them and other groups in the past twenty years. A sugar reporter capable of entering the metabolic pathway under scrutiny and equipped with a reactive handle is first introduced in a living organism of interest, followed by the bioorthogonal ligation of an appropriately functionalized probe, most often a fluorescent dye for optical bioimaging.^15^ These methods rely on biocompatible click reactions that involve π_4_s + π_2_s pericyclic processes, namely the copper-catalysed azide–alkyne (3+2) cycloaddition, the strain-promoted azide–alkyne (3+2) cycloaddition and the inverse electronic demand Diels–Alder reaction (CuAAC, SPAAC and IEDDA, respectively). Indeed, such mechanisms are mostly absent from living systems, thus greatly minimizing interference with biological processes.^16^

Although many bioorthogonal methods have been used in prokaryotic cells to track proteins with non-canonical amino acids,^17^ efforts to apply MOE to detect glycans in prokaryotes remain rather meager by comparison, as these strategies were developed in human models first and foremost. Some glycoengineering methods were reported in various species to label bacterial glycans, which were extensively reviewed by Banahene *et al*.^18^ They aim at detecting components of bacterial cell walls, such as peptidoglycans with *N*-acetylmuramic acid derivatives^19,20^ or lipopolysaccharides with, for example, azide-modified analogues of *N*-acetylglucosamine,^21^ 3-deoxy-d-manno-octulosonic acid^22^ or legionaminic acid precursors.^23^ However, very few bacterial species have been specifically labelled on their capsular polysaccharides by bioorthogonal chemistry. We could only identify two reports, in which capsules of commensal bacteria of the *Bacteroides* genus have been effectively tagged by MOE with modified *N*-acetylgalactosamine reporters.^24,25^ The authors visualized the capsular component of prelabelled bacteria in live mice intestines, opening an avenue to better understand host–pathogen interactions and the evolution of related diseases.

To the best of our knowledge, capsular polysaccharides constituted of sialic acids have thus never been investigated by MOE in combination with click chemistry, despite the importance of these structures as virulence factors. Herein, we report a bioorthogonal labelling method to detect the polysialic acid capsule using alkyne- and azide-modified ManNAc and Neu5Ac reporters in *E. coli* K1 strains. Although this reporter toolbox is commonly used in mammalian systems to label *N*-glycoproteins,^26,27^ it is the first time that these reporters are used successfully in *E. coli*. We aimed to take advantage of the fact that, unlike non-pathogenic strains, K1 strains are capable of synthesizing Neu5Ac *de novo*, and developed a test that allows facile and specific detection of the PSA capsule. The method was miniaturized into a microplate assay for transferability to screening approaches, which could serve as an interesting platform to decipher K1 pathways and identify external factors that influence PSA biosynthesis. Such assays could help in the discovery of new capsule-targeted classes of antibiotic drugs that decrease the virulence of a pathogenic bacterium while circumventing known resistance mechanisms.

## Results and discussion

### Chemical reporters

Per-*O*-acetylated monosaccharides are commonly used for MOE in mammalian models. However, per-*O*-acetylation can cause issues as it may generate non-specific signal and false positives.^28,29^ It may also be conducive to acidification of the intracellular milieu, metabolic perturbation due to partially acetylated forms, or cytotoxicity.^16^ In addition, it has been suggested that low levels of non-specific esterase activity prevent the efficient release of the free sugar form thus thwarting the use of such reporters in some prokaryotic species including *E. coli*.^23^ Conversely, this species is capable of active transport mediated ManNAc uptake *via* the ManXYZ transporter and can also scavenge exogenous sialic acid from the host *via* the sialic acid transporter NanT. We therefore decided to use unprotected reporters and synthesized *N*-(2-azidoacetyl)-d-mannosamine (ManNAz), *N*-4-pentynoyl-d-mannosamine (ManNAl), *N*-(2-azidoacetyl)-neuraminic acid (SiaNAz) and *N*-4-pentynoylneuraminic acid (SiaNAl) (see experimental section). CuAAC was chosen as the bioorthogonal reaction, owing to its fast kinetics and ease of use. In addition, the azide and terminal alkyne tags are easily interchanged thus allowing comparison of the chemical handle's impact.

### CuAAC whole cell labelling of K1 expressing *E. coli* EV36

**Fig. 2:**
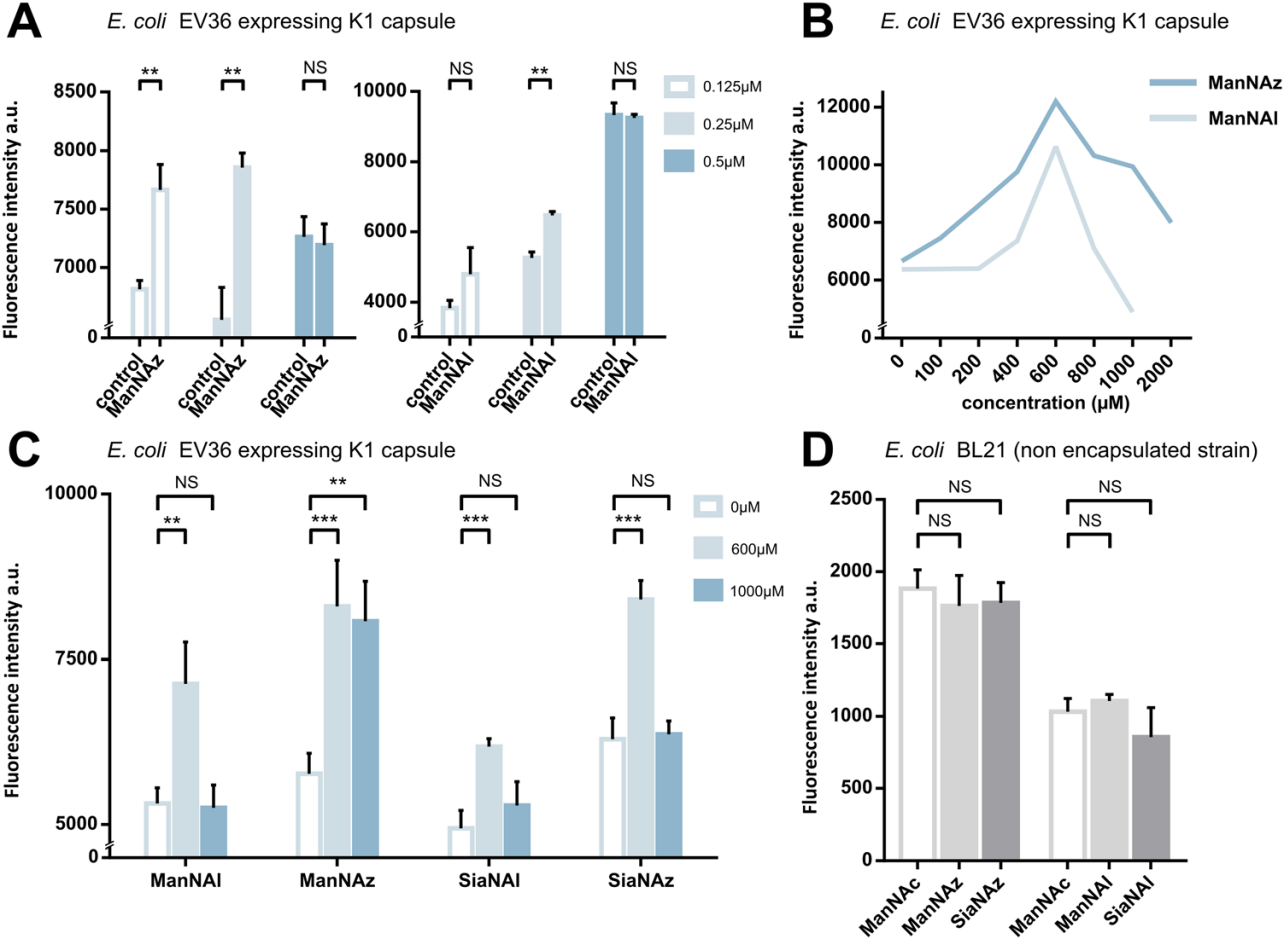
Whole cell suspension fluorescence after MOE in *E. coli* EV36. (A) Optimization of the fluorophore concentration. *E. coli* EV36 were grown with 1 mM ManNAz or 1 mM ManNAl, followed by CuAAC ligation of TAMRA-Alk or TAMRA-N_3_, respectively, at concentrations of 0.125, 0.25 μM or 0.5 μM. Negative control samples were grown with 1 mM ManNAc (Ctrl) and reacted in the same conditions. (B) Optimization of the chemical reporter concentration. *E. coli* EV36 were grown with ManNAl or ManNAz at concentrations ranging from 0 to 2000 μM. The CuAAC ligation was then performed using the relevant fluorophore (TAMRA-N_3_ or TAMRA-Alk, respectively) at a concentration of 0.25 μM. (C) Miniaturized assay on *E. coli* EV36 in 96-well plates. CuAAC labelling was performed using the identified optimal conditions, namely 0.25 μM dye for 45 minutes in presence of the catalytic buffer. (D) Negative control experiments carried out on *E. coli* BL21, a non-encapsulated strain. 3 biological replicates with a minimum of 3 technical replicates were measured. Results are presented as mean + SD. Unpaired *t*-tests were performed to get statistical significance of difference observed compared to control condition (NS = non significant (*P* > 0.05); ** = *P* < 0.01; *** = *P* < 0.001).

We first assessed whether these reporters could be taken up and metabolically incorporated into living *E. coli* EV36, a strain containing all fourteen genes of the *kps* PSA biosynthetic cluster that produces the K1 capsule.^30–32^ EV36 cultures were incubated overnight in lysogeny broth (LB) medium complemented with the modified monosaccharides, prior to CuAAC labelling with an appropriate clickable tetramethylrhodamine dye bearing an alkyne or azide reactive moiety (TAMRA-N_3_ for alkynyl reporters and TAMRA-Alk for azido reporters). The same fluorochrome was used in both instances in order to allow intensity-based comparisons. The reactive function is not π-conjugated to the rhodamine core owing to the presence of an oligoPEG spacer arm, and both dyes present the same photophysical characteristics (*Φ* = 0.1; *ε* = 92.000 M^−1^ cm^−1^, superimposable spectra). The assay was implemented using a fluorescence microplate reader to enable facile testing of multiple conditions. The bioorthogonal ligation was first optimized to obtain a robust labelling and satisfying signal-to-noise ratio in EV36 whole cells. Various parameters were screened, with TAMRA dye concentrations ranging from 62.5 nM to 25 μM in EV36 cultures incubated with reporters at a fixed 1 mM concentration, giving the best signal to noise ratio at 0.25 μM of fluorophore for 45 minutes (Fig. 2A), and then with chemical reporter concentrations ranging from 100 μM to 2 mM at a fixed 0.25 μM TAMRA concentration, resulting in an optimal chemical reporter concentration found at 600 μM (Fig. 2B). In contrast to what has been reported in activity-based protein profiling studies in mammalian cell lysates using dye concentrations 200 to 400 fold higher,^33,34^ we observed a higher level of background using TAMRA-N_3_ at 0.5 μM. This could be attributed to non-covalent interactions of azide dipoles at the bacterial cell surface at higher concentrations, or to the fact that alkyne-dyes exert less non-specificity in the nanomolar range.

Next, we sought to perform statistically robust tests to determine the significance of the difference in labelling after the incorporation of the chemical reporters at 600 μM and 1 mM. In order to allow transferability of the protocol to prospective screening approaches, we developed this assay in 96-well plates, using an intensity-based readout with a fluorescence microplate reader. For all four compounds, we observed significant labelling at 600 μM but not at 1 mM, except for ManNAz being the only analogue where the labelling was significant in both cases (Fig. 2C). This suggested that ManNAl, SiaNAl and SiaNAz might exert a negative effect on the physiological state or viability of bacteria at higher concentrations.

Experiments in which the bacteria were labelled with SiaNAz and SiaNAl generally exhibited a lower fluorescence intensity when compared to ManNAz and ManNAl, which came as a surprise. Indeed, these microbial cells are generally thought to be able to scavenge, process and install Sia derivatives but to metabolize ManNAc very slowly,^35,36^ although this is highly species dependent. ManNAz led to the most efficient labelling. The noticeable difference in intensity between ManNAz and ManNAl tagging indicates that the azide handle may be more compatible with the metabolic network overall, or that the alkyne moiety might induce toxicity during the metabolic incorporation step. Distinct uptake and/or metabolisms should account for differences in labelling efficiency between ManNAc or Sia derivatives bearing the same chemical tag (*e.g.*, ManNAz and SiaNAz).^36^

### Reporter accumulation in the capsule

**Fig. 3:**
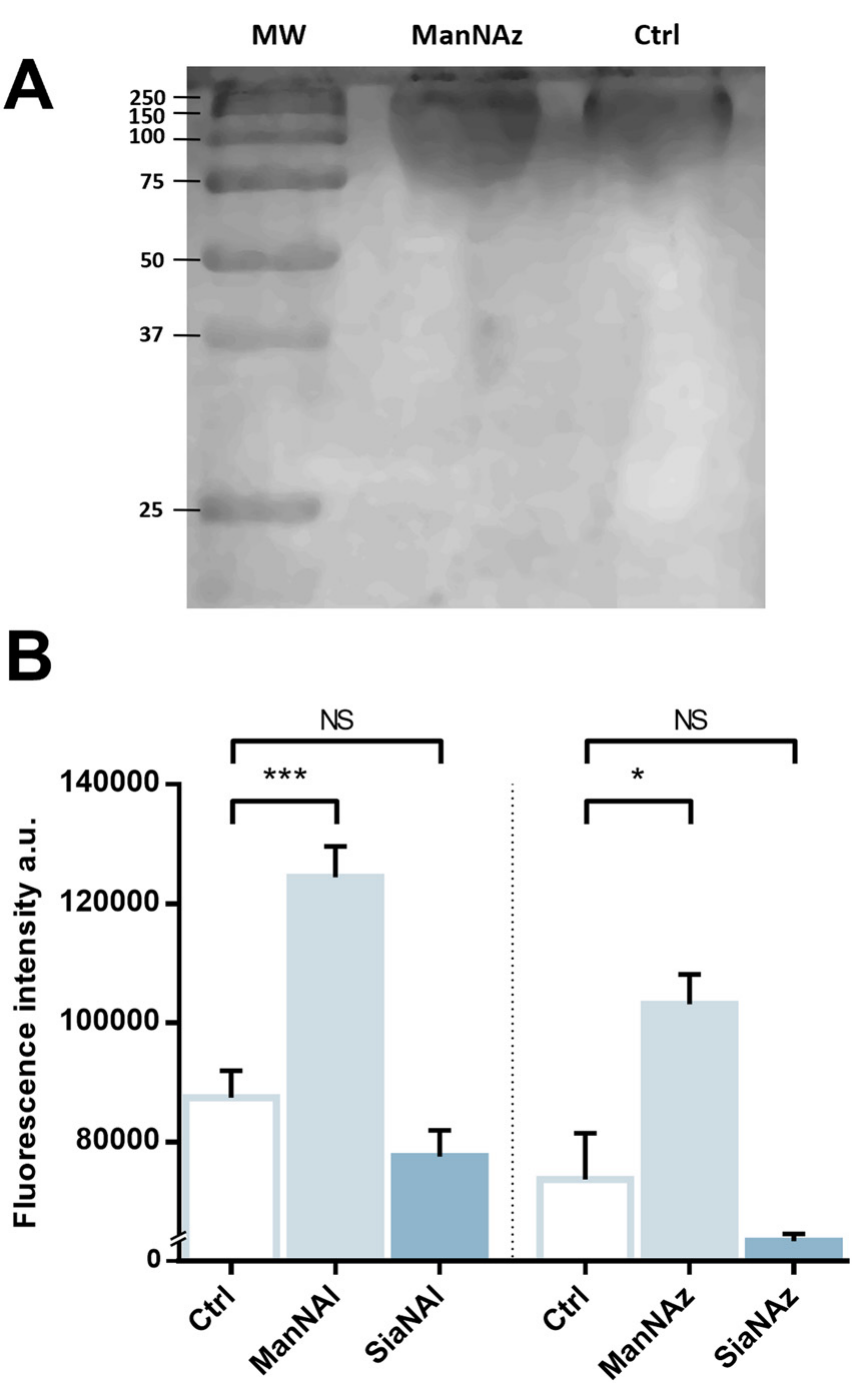
Capsular extraction and fluorescence quantification – (A) The capsular polysaccharides were purified after metabolic incorporation and analysed by PAGE with alcian blue staining. Molecular weight (MW) standard is indicated in kDa. (B) Capsular extracts obtained after metabolic incorporation of chemical reporters were labelled by CuAAC with 5 μM TAMRA-N_3_ or TAMRA-Alk. 3 biological replicates with a minimum of 3 technical replicates were measured. Results are presented as mean +/− standard deviation. Unpaired *t*-tests were performed to get statistical significance of the difference measured compared to the control condition (Ctrl). NS = non significant (*P* > 0.05); * = *P* < 0.05; *** = *P* < 0.001.

We pursued by investigating whether the observed labelling originates from reporters incorporated into the K1 capsule. To show this, *E. coli* EV36 was grown in LB medium complemented with ManNAz or ManNAc, then the K1 capsular polysaccharides were extracted and purified according to a previously described procedure.^37^ A polyacrylamide gel electrophoresis of the extracted K1 capsule was performed, followed by alcian blue staining (a dye that specifically reveals acidic polysaccharides^38^) to confirm the presence and purity of PSA (Fig. 3A). To evaluate the incorporation of the chemical reporters in the isolated capsule, the extracted acidic polysaccharides were then labelled in solution by CuAAC and fluorescence intensity was measured as described hereabove. Specific labelling was observed with both ManNAc derivatives ManNAl and ManNAz, but not with the Neu5Ac analogues SiaNAl and SiaNAz (Fig. 3B). Therefore, this indicates that chemical reporters ManNAz and ManNAl enter the bacterial cell (possibly through the ManXYZ-encoded transporter) and are converted to SiaNAz and SiaNAl, respectively, in the bacterial cytoplasmic compartment.^39^ The derived tagged Sia is subsequently activated as CMP-Sia, polymerized and exported at the cell surface by various enzymes and transporters.^9^*E. coli* has been described to convert ManNAc to ManNAc-6-P during uptake to redirect the product on the degradation pathway.^39,40^ Our results suggest that *E. coli* can also incorporate ManNAc into the K1 capsule biosynthesis *via* an unidentified pathway able to either transport ManNAc *via* the manXYZ phosphotransferase system, producing intracellular ManNAc-6-P which is then converted to ManNAc by an unknown phosphatase, or to transport ManNAc without ManNAc-6-P conversion. The former hypothesis carries a much stronger weight as the presence of this enzyme was previously suggested in *Neisseria meningitidis*, which also expresses K1 capsules, though no proper characterization has yet been reported.^41^ Furthermore, Neu5Ac is transported by the NanT sialate permease to the interior of the bacterial cells and is known to be directly degraded.^30,35,40,42,43^ Our results are in good agreement with this observation. Neu5Ac is indeed transported inside the cell as demonstrated by the whole cell fluorescence observed (Fig. 2), but the monosaccharide is not directed to the PSA biosynthesis as suggested by the lack of signal detected on the purified capsule (Fig. 3B).

To check whether constitutive biomolecules other than the capsule were labelled by our strategy, we carried out the same experiments on whole *E. coli* BL21 cells, a B strain that does not express capsules. All four reporters were tested, and no labelling was observed on this strain (Fig. 2D), thereby confirming the specific incorporation of the reporters into the K1 PSA capsule in the EV36 bacterium.

### Effect of unnatural monosaccharides on the K1 capsule biosynthesis

**Fig. 4:**
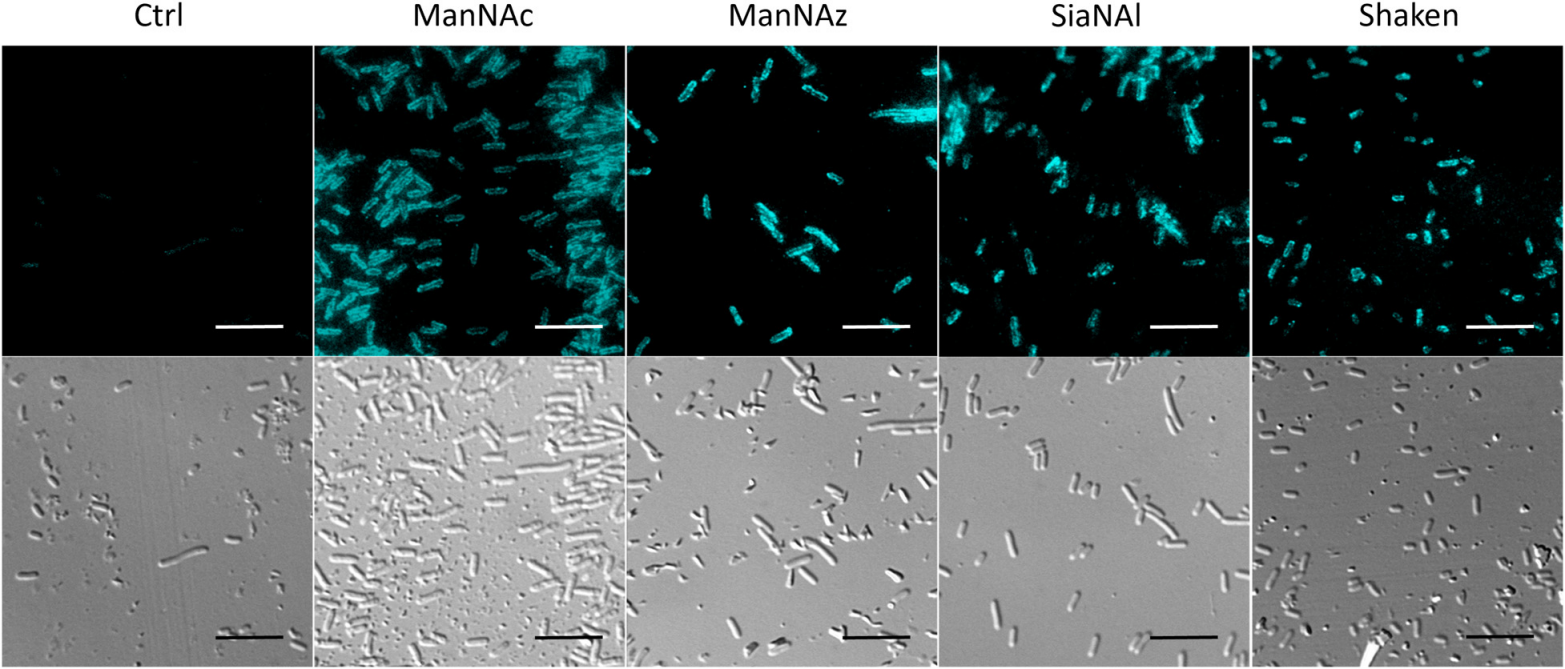
Immunofluorescence microscopy of the K1 capsule from *E. coli* EV36 with antibodies. *E. coli* EV36 bacteria were grown with either ManNAc, ManNAz, or SiaNAl at 1 mM before being incubated with anti-K1 rabbit IgG antibody ENZ-ABS559-0100 recognizing homopolymers of α2,8-linked sialic acid of *E. coli* K1 (1/100, 45 min), rinsed with 200 μl PBS and treated with Alexa Fluor 488 conjugated anti-rabbit secondary antibody (1/250, 30 min). *Top*: fluorescence channel. *Bottom*: Differential interference contrast (DIC) channel. Scale bar = 10 μm. For the shaken condition, cells were submitted to the same shaking and rinsing conditions needed for CuAAC to confirm that the K1 capsule is still intact and not washed away after these treatments. For the control condition (Ctrl), the secondary antibody was omitted to get the autofluorescence background.

To ensure that the capsule was correctly expressed in the different labelling conditions, we performed immunofluorescence labelling of whole *E. coli* EV36 cells with an anti-K1 antibody followed by observation in fluorescence microscopy. A neat peripheral labelling pattern was observed, confirming the presence and integrity of capsular PSA. The experiments were carried out after growth in the presence of a ManNAc or Neu5Ac analogue (ManNAz and SiaNAl, respectively), or after the thorough rinsing and shaking conditions required during the CuAAC labelling protocol (“Shaken” condition) (Fig. 4). These results demonstrate a robust expression of the K1 capsule, without side effects of the unnatural monosaccharides on the K1 pathways.

### Impact of unnatural monosaccharides on the growth and physiological state of the bacteria.

**Fig. 5:**
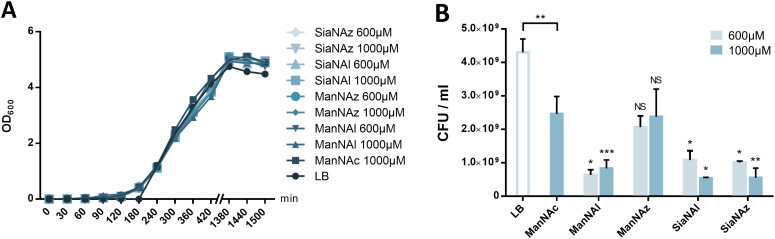
Growth and viability of *E. coli* EV36 in the presence of unnatural monosaccharides. (A) Growth assay of *E. coli* EV36 cultures with different chemical reporters at 0.6 mM and 1 mM compared to the control conditions (LB). (B) Viability assay of *E. coli* EV36 cultures after growth with different chemical reporters at 0.6 mM and 1 mM. See experimental part for details. 3 biological replicates with a minimum of 3 technical replicates were measured. Results are presented as mean + SD. Unpaired *t*-tests were performed to get statistical significance of difference observed compared to ManNAc 1 mM condition (above error bars), as well as between ManNAc 1 mM and LB (above brackets) (NS = non significant (*P* > 0.05); * = *P* < 0.05; ** = *P* < 0.01; *** = *P* < 0.001).

To further investigate the results obtained in Fig. 2C, *E. coli* EV36 growth and viability were evaluated for ManNAz, ManNAl, SiaNAz, or SiaNAl at 1 mM and 0.6 mM in comparison to ManNAc and LB alone. To evaluate the impact of the chemical reporters on the bacterial growth we measured the optical density at 600 nm (OD_600_) of LB suspensions at regular intervals over the course of several hours. No difference was observed between the various conditions, illustrating that the reporters do not impair cell growth (Fig. 5A). To evaluate the impact on long-term cell viability, serial dilutions of the suspensions were carried out after growth that were plated for viable counts. All chemical reporters except ManNAz induced rather significant toxicity on the long term when compared to ManNAc. Interestingly, when ManNAc was added to the medium the cell viability was also significantly lower by comparison to LB medium alone (Fig. 5B). ManNAz and ManNAc equally affect bacterial long-term viability to a mild extent. On the contrary, ManNAl drastically reduces the viability at both concentrations, revealing an increased toxicity linked to the appended pentynoyl moiety. This toxicity leads to decreased levels of specific PSA labelling (Fig. 2), and might enhance non-specific binding. Dead bacteria have been recently described to form nanotubes by membrane extrusion or vesicles. These structures may impact background levels.^44^ Such an effect due to the nature of the bioorthogonal handle has been evidenced before in human Jurkat cells, where azidofucose derivatives proved to be more efficiently incorporated but more toxic than their alkynyl counterparts.^45^ In the case of K1-expressing *E. coli*, it is the alkyne tag that decreases the viability of EV36 cells, which might be explained by the fact that a lower level of incorporation in capsular PSA leads to increased catabolism and accumulation of alkyne by-products interfering with other metabolic networks in the cell. Nevertheless, ManNAl is efficiently incorporated into the capsule (Fig. 3B).

It could thus be hypothesized that the pentynoyl sidechain is less compatible with the *E. coli* NeuB-encoded sialic acid synthase or NeuA-encoded CMP-sialic acid synthase than its 2-azido counterpart in ManNAz. This result calls attention to the use of unnatural monosaccharides to study bacterial physiology. To avoid any misinterpretation, viability assays should always be performed when testing MOE strategies on a new bacterial strain.

### MOE microscopy

**Fig. 6:**
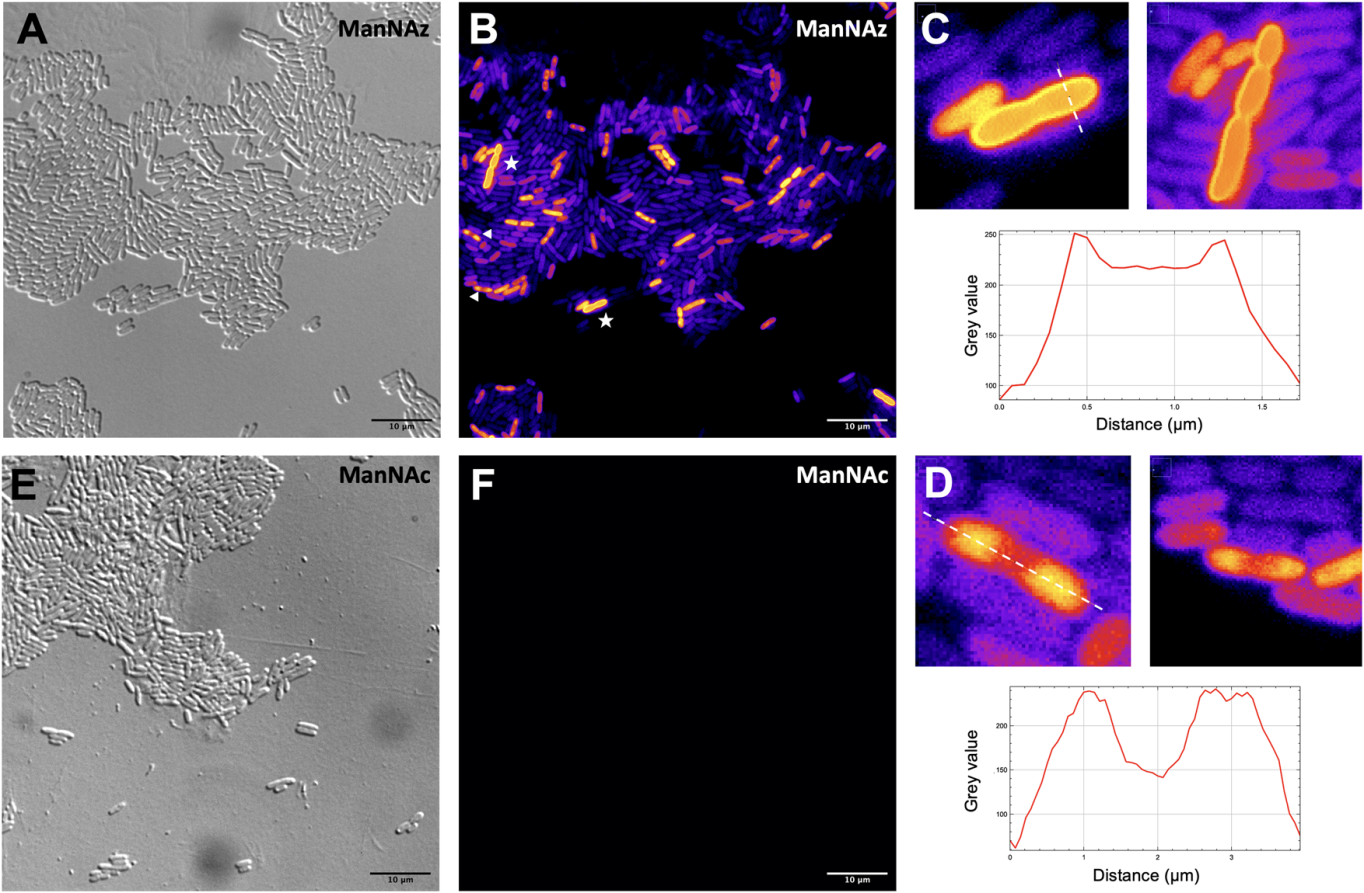
Fluorescence microscopy of *E. coli* EV36 after incorporation of ManNAz at 600 μM. CuAAC was performed with the TAMRA-Alk dye at 100 mM. Brightfield channel (A) depicted in greyscale, fluorescence channel (B and F) depicted in “Fire” LUT colorscale. (C) (top) Magnified zooms on cells showing a pericellular labelling (highlighted by white stars in insert B) and (bottom) intensity profile along the transversal dashed line; (D) (top) Magnified zooms on cells showing a polar labelling (highlighted by white triangles in insert B) and (bottom) intensity profile along the longitudinal dashed line. (E and F) Negative control incubated with ManNAc and reacted in the same CuAAC conditions (TAMRA-Alk, 100 mM). Experiments were carried out as 3 biological replicates. Scale bars= 10 μm.

Given that ManNAz enables specific labelling of the capsule with no significant toxicity nor impact on growth, we chose to use this analogue for MOE followed by CuAAC labelling of the capsule for observation by fluorescence microscopy. After ManNAz incorporation, cells were treated with a CuAAC buffer whose composition was adapted from a previously reported procedure.^22^ A bright, heterogeneous and specific labelling was observed after ManNAz incorporation compared to ManNAc as negative control (Fig. 6). All cells appear to incorporate the reporter. In most cells, the labelling is of rather low intensity (although significant when compared to the control) but on some others, a bright peripheric fluorescent signal was visible (Fig. 6B and C), typical of capsule localisation. Additionally, a number of cells exert a labelling pattern at the poles of the bacterium (Fig. 6D), and most cells that do appear to be undergoing or to have just undergone cell division. This could be interpreted as a physiological state in which the older labelled PSA capsule has been relocated at the polar regions while new unlabelled capsule is being produced at the septum region during binary fission. Like most capsular polysaccharides in Gram-negative bacteria, PSA assembly and export through the periplasm is controlled by a multiprotein complex. The K1 antigen belongs to group 2, which identifies strains for which this export involves an ABC-transporter.^46^ Our results might indicate that this translocation to the outer membrane is spatially regulated at the equatorial region during cell division, similarly to what has been proposed for Gram-positive *Streptococcus pneumoniae*, whose capsule export is mediated by the Wzx–Wzy system.^47^ Indeed, Henriques *et al.* suggested that this spatial control ensures the coordination of capsule and cell wall biogenesis at the division site, leaving the bacteria unexposed to the host's immune system throughout. The heterogeneous labelling points out the incorporation of ManNAz with a potential dependence on the physiological state of the bacteria during their growth. Similarly, the bacteria need to adjust their metabolism to ManNAc and ManNAz import and successfully export the polysialic acid at the cell surface, which may also result in heterogeneity. Finer localisation may be obtained with super-resolution microscopy, to refine the visualization of capsule export zones at the poles of the cells.^48^

**Fig. 7:**
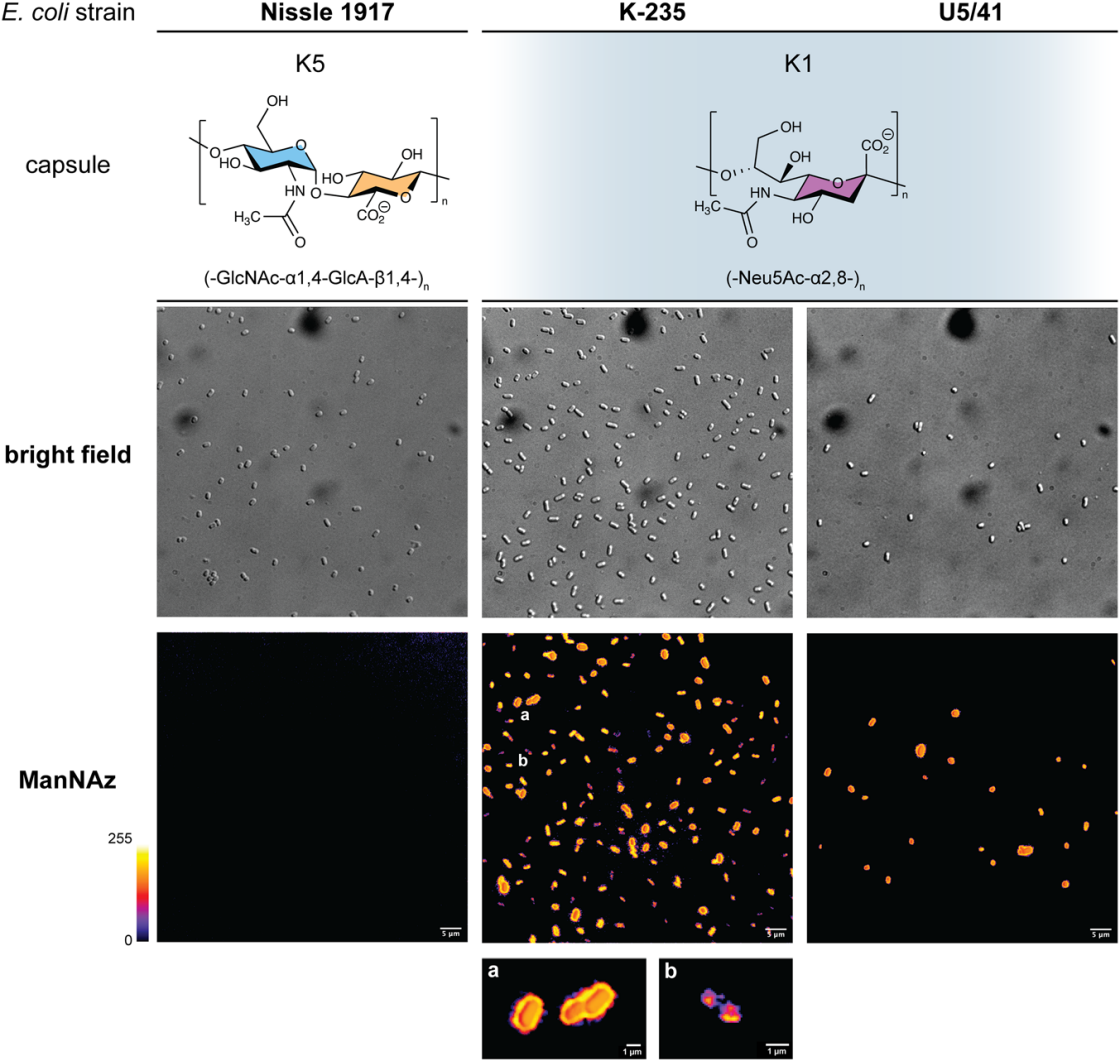
Fluorescence microscopy of *E. coli* strains Nissle 1917 expressing the K5 capsular polysaccharide (heparosan), and K-235 and U5/41 expressing the K1 capsular polysaccharide (PSA), grown with ManNAz at 600 μM overnight then labelled with TAMRA-Alk – (top) brightfield channel; (bottom) fluorescence channel in Fire colorscale LUT. (a) zoom on bacteria exhibiting a peripheral pattern typical of capsule expression, (b) zoom on bacterium with labelling in the polar regions during cell division. Experiments were carried out as 3 replicates. Scale bar = 5 μm.

In order to further confirm the specificity of our method for the K1 capsule and ascertain its applicability to various pathogenic bacteria, we then tested three other wild-type *E. coli* strains (Fig. 7). In addition to *E. coli* BL21,^49^ a strain devoid of capsule mentioned hereabove as negative control (Fig. 2D), we also evaluated the ability of *E. coli* Nissle 1917 bacteria to metabolize ManNAz. The Nissle 1917 strain expresses another capsular acidic polysaccharide, namely the K5 antigen heparosan constituted of a disaccharide repeating unit that contains glucuronic acid and *N*-acetylglucosamine.^8,50^ As expected, this strain was unable to incorporate ManNAz, further confirming the specificity of this assay for the K1 PSA capsule. Moreover, the two *E. coli* K1 pathogenic strains K-235 and U5/41, which produce significantly higher levels of PSA than the EV36 model, exhibited strong fluorescence (Fig. 7). This indicates an increased level of incorporation of SiaNAz units into PSA derived from the metabolization of ManNAz when compared to experiments on EV36. The heterogeneity was lesser in the K-235 and U5/41 wild-types and the pattern observed was consistent, with bacteria marked at the periphery typical of capsule staining and other bacteria showing localization at the poles. In contrast, the heterogenous incorporation observed in the EV36 construct might be attributed to a less efficient metabolic channeling process, induced by the expression of exogenous genes from K1 strains in a K-12 background (*e.g.*, the manXYZ operon). The presence of PSA in the K-235 and U5/41 strains and its absence in the Nissle 1917 strain were further confirmed by immunofluorescence using a fluorescent anti-K1 antibody (Fig. S1, ESI†). Thus, our method can be used to detect K1 antigen production in pathogenic strains, which could help decipher the dynamics of capsule expression and the factors that regulate it in future studies. Furthermore, it provides a platform for screening new types of antibiotics targeting K1 capsule metabolism.

## Experimental

### Chemical synthesis of unnatural monosaccharides

ManNAl (*N*-4-pentynoyl-d-mannosamine): 4-pent-ynoïc acid (502 mg, 5.1 mmol, 1 eq.) was solved into CH_2_Cl_2_ (25 ml). *N*-Hydroxysuccinimide (674 mg, 5.8 mmol, 1.14 eq.) and EDC or 1-ethyl (3-dimethylaminopropyl)carbodiimide (1.96 g, 10.2 mmol, 2 eq.) were slowly added. The mixture was stirred for 3 h 30 at room temperature. The end of reaction was confirmed by TLC (cyclohexane/AcOEt; 70 : 30). The solution was then washed four times with KHSO4 (aqueous solution 2.8%). The recombinated organic layers were dried over sodium sulfate, filtered, and then concentrated under reduced pressure to give the succinimidyl ester as a white solid (875.3 mg, 4.48 mmol, 88%). The product was used in the next step without further purification. The coupling reaction with Mannosamine hydrochloride was conducted under nitrogen atmosphere. Hydrochloride d-Mannosamine (759 mg, 3.52 mmol, 1 eq.) and the succinimidyl ester from previous step (688 mg, 3.52 mmol, 1 eq.) were solved into DMF (25 ml). Triethylamine (1.4 ml, 10.77 mmol, 3 eq.) was slowly added. The mixture was stirred overnight. DMF was then removed under reduced pressure and the crude product was purified by flash chromatography (silica column, 40 g, 30 μm) with an elution by CH_2_Cl_2_/EtOAc/MeOH; 45 : 45 : 10). Solvents were then eliminated to yield ManNAl as a white solid (830 mg, 3.34 mmol, 95%). Mixture of anomers (*α*/*β* ≈ 60%/40%).

α ^1^H NMR (300 MHz, D_2_O): *δ* = 5.00 (d, *J* = 1.4, 1H, H_1_), 4.23 (dd, *J* = 4.7, 1.4, 1H, H_2_), 3.93 (dd, *J* = 9.8, 4.7, 1H, H_3_), 3.80 – 3.62 (m, 3H, H_5_ & H_6_), 3.50 (t, *J* = 9.6, 1H, H_4_), 2.58 – 2.32 (m, 4H, H_8_ & H_9_), 2.26 (t, *J* = 2.3, 1H, H_11_).

^13^C NMR (75 MHz, D_2_O): *δ* = 175.26 (C7), 93.17 (C1), 83.43 (C10), 71.94 (C5), 70.14 (C11), 68.73 (C3), 66.72 (C4), 60.36 (C6), 53.15 (C2), 34.10 (C8), 14.40 (C9).

β ^1^H NMR (300 MHz, D_2_O): *δ* = 4.90 (d, *J* = 1.6, 1H, H_1_), 4.35 (dd, *J* = 4.4, 1.4, 1H, H_2_), 3.82 – 3.61 (m, 3H, H_3_ & H_6_), 3.40 (t, *J* = 9.8, 1H, H_4_), 3.29 (ddd, *J* = 9.9, 4.8, 2.3, 1H, H_5_), 2.53 – 2.31 (m, 4H, H_7_ & H_8_), 2.26 (t, *J* = 2.3, 1H, H_11_).

^13^C NMR (75 MHz, D_2_O): *δ* = 175.98 (C7), 92.84 (C1), 83.85 (C10), 76.28 (C5), 71.96 (C3), 69.99 (C11), 66.45 (C4), 60.36 (C6), 53.98 (C2), 34.25 (C8), 14.26 (C9).

SiaNAl (*N*-4-pentynoylneuraminic acid): ManNAl, (60 mg, 0.23 mmol, 1 eq.), sodium pyruvate (46 mg, 0.345 mmol, 2 eq.) and Neuraminic5Ac aldolase (Sigma Aldrich EC 4.1.3.3) from E. Coli K12 (5 units dissolved in 0.1 ml) were added into 600 μL of phosphate buffer (KH_2_PO_4_, 10.3 mM, K_2_HPO_4_, 52.8 mM and MgCl_2_ 20 mM, pH – 7.5) into a reaction tube which was incubated at 37.5 °C with moderate shaking (140 rpm) for 18 hours. The reaction completion was monitored by TLC (propan-1-ol/25% Ammonia/H_2_O; 6 : 1: 2.5). Reaction was quenched by the addition of 30 ml of water. The product was purified on an anion exchange resin (Bio-Rad AG1X2) activated with NH_4_HCO_3_ 0.1 M. The sample was loaded on the column which was washed with H2O (5 CV). Elution was performed with aqueous NH_4_HCO_3_ (0.05 M, 5 CV) and then NH_4_HCO_3_ (0.2 M, 5 CV). Fractions were detected by TLC (same elution as for the reaction monitoring) combined and freeze dried. After being solved into H_2_O (1 ml), sample was passed through a gel filtration column (P2) and then freeze dried again. SiaNAl was obtained as white powder (73 mg, 0.21 mmol, 91%).

^1^H NMR (600 MHz, D_2_O): *δ* = 3.96 – 3.86 (m, 2H, H_4_ & H_9_), 3.83 (t, *J* = 10.1, 1H, H_5_), 3.72 (dd, *J* = 11.9, 2.7, 1H, H_9_), 3.64 (ddd, *J* = 9.2, 6.7, 2.7, 1H, H_8_), 3.51 (d, *J* = 9.2, 1H, H_7_), 3.49 – 3.43 (m, 1H, H_9′_), 2.47 – 2.34 (m, 4H, H_11_ & H_12_), 2.28 (d, *J* = 2.0, 1H, H_13_), 2.10 (dd, *J* = 13.0, 4.9, 1H, H_3eq_), 1.70 (dd, *J* = 12.7, 11.7, 1H, H_3ax_).

^13^C NMR (75 MHz, D_2_O): *δ* 175.43 (C1), 172.91 (C10), 95.16 (C2), 83.54 (C13), 70.40 (C14), 70.37 (C8), 70.23 (C6), 68.29 (C7), 66.45 (C4), 63.21 (C9), 52.04 (C5), 38.79 (C3), 34.67 (C11), 14.53 (C12).

*m*/*z*: Calculated: [M]+ = 346.312; measured: 346.017.

ManNAz (*N*-azidoacetyl-d-mannosamine): synthesis, 2-azidoacetic acid (117 mg, 1.16 mmol, 1 eq.) was dissolved along with DIC (*N*,*N*′-diisopropylcarbodiimide, 175 mg, 1.392 mmol, 1.2 eq.), HOBt (hydroxybenzotriazole, 195 mg, 1.276 mmol, 1.1 eq.) and DIPEA (*N*,*N*-diisopropylethylamine, 141 mg, 1.392 mmol, 1.2 eq.) in DMF (dimethylformamide, 20 ml), d-mannosamine hydrochloride (250 mg, 1.16 mmol, 1 eq.) was added and the reaction was stirred at room temperature for 19 h under argon. The total consumption of mannosamine was assessed by silica thin layer chromatography (TLC) (CH_2_Cl_2_/MeOH 9 : 1 v/v) before solvent removal under reduced pressure. The reaction crude was purified by silica flash column chromatography (50 μm, 40 g, dry load, CH_2_Cl_2_/MeOH 95 : 5 v/v), and fractions containing the product were gathered and concentrated under reduced pressure, affording the ManNAz as a white powder (175 mg, 0.67 mmol, 57%).

^1^H NMR (300 MHz, D_2_O): *δ* = 5.10 (d, *J* = 1.0 Hz, 1H, H_1_ α), 5.01 (d, *J* = 1.4 Hz, 1H, H_1_ β), 4.46 (dd, *J* = 4.0 Hz, 1.4Hz, 1H, H_2_ β), 4.33 (dd, *J* = 4.4 Hz, 1.0 Hz, 1H, H_2_ α), 4.10 – 3.98 (m, 5H, H_3_ α + H_8_ αβ), 3.88 – 3.71 (m, 5H, H_5_ α + H_3_ β + H_6_ αβ), 3.56 (t, *J* = 9.5Hz, 1H, H_4_ α), 3.46 (t, *J* = 9.8Hz, 1H, H_4_ β), 3.38 (ddd, *J* = 9.8Hz, 4.7Hz, 2.1Hz, 1H, H_5_ β).

^13^C NMR (75 MHz, D_2_O): *δ* = 171.87 (s, C7 α), 170.97 (s, C7 β), 92.89 (s, C1 α), 92.83 (s, C1 β), 76.40 (s, C5 β), 72.03 (s, C3 β), 71.97 (s, C5 α), 68.82 (s, C3 α), 66.76 (s, C4 α), 66.52 (s, C4 β), 60.41 (s, C6 β), 60.39 (s, C6 α), 54.28 (s, C2 β), 53.37 (s, C2 α), 51.72 (s, C8 β), 51.65 (s, C8 α).

SiaNAz (*N*-azidoacetylneuraminic acid): ManNAz (42 mg, 0.159 mmol, 1 eq.) was dissolved in 600 μl PBS (pH 7.6) with sodium pyruvate (32 mg, 0.239 mmol, 1.5 eq.) in a 5 ml capped tube. 5 units of Neu5Ac aldolase (Sigma Aldrich, EC 4.1.3.3) were added and the reaction was stirred at 37 °C for 16 h. The formation of the product was checked by TLC (1-propanol, NH_3_, H_2_O 6 : 1 : 2.5) with resorcinol as a reagent. The reaction crude was purified by anion exchange chromatography (BioRad, AG1X8; H_2_O 5 CV, NH_4_HCO_3_ 0.05 M 5 CV, NH_4_HCO_3_ 0.2 M 5 CV). Fractions containing the product were identified by TLC as previously then gathered and concentrated under reduced pressure. The white powder obtained was desalted using size exclusion chromatography (P2) with ultrapure water as eluent. Fractions containing the product were gathered, concentrated under reduced pressure then freeze-dried, affording the product as a white powder (18.5 mg, 0.053 mmol, 34%).

^1^H NMR (300 MHz, D_2_O): *δ* 4.09–3.88 (m, 5H, H_5_ + H_4_ + H_3_ + H_2_), *δ* 3.78 (dd, *J* = 11.2Hz, 2.2Hz, 1H, H_8cis_), *δ* 3.65 (ddd, *J* = 8.5Hz, 4.1Hz, 1.2Hz, 1H, H_7_), *δ* 3.55 (dd, *J* = 11.2Hz, 5.8Hz, 1H, H_8trans_), *δ* 3.45 (dd, *J* = 9.2Hz, 0.9Hz, 1H, H_6_), δ 2.18 (dd, *J* = 12.5 Hz, 4.8Hz, 1H, H_1_ eq.), *δ* 1,75 (dd, *J* = 12.4Hz, 11.8Hz, 1H, H_1_ ax)

^13^C NMR (75 MHz, D_2_O): *δ* 70.8 (CH, C5), *δ* 69.82 (CH, C7), *δ* 68.43 (CH, C6), *δ* 67.01 (CH, C2), *δ* 63.10(CH2, C8), *δ* 52.21(CH, C3), *δ* 51.9 (CH2, C4), *δ* 39.32 (CH2, C1)

### Bacterial strains and growth conditions

Five bacterial strains were used in this study. The non-pathogenic model *E. coli* EV36^30^ that expresses the K1 capsule was kindly provided by Pr Antonia P. Sagona. The K1 producing pathogenic strains *E. coli* K-235 (ATCC 13027) and *E. coli* U5/41 (ATCC 11775) were purchased from ATCC. *E. coli* BL21,^49^ a well-described B strain, and *E. coli* Nissle 1917 expressing the K5 capsule were used as negative controls to demonstrate the specificity of the method for K1 capsule and were kindly provided by Dr Marie Titecat. The bacteria were streaked out from −80 °C stocks on LB agar Petri dishes and cultured overnight at 37 °C. Isolated colonies grown on these plates were used to inoculate liquid cultures for the rest of the experiments.

### Metabolic incorporation and CuAAC buffer preparation

*E. coli* liquid cultures prepared as explained above were diluted to an OD_600_ of 1. 20 μl of this solution was transferred to 10 ml LB (supplemented with the desired chemical reporters) in a 50 ml conical tube and grown overnight at 37 °C/180 rpm. For fluorescence labelling by CuAAC, fresh aqueous stock solutions of the different reagents were used to prepare the CuAAC buffer. It should be prepared several minutes to an hour prior to use and should not be kept for more than a few hours. CuSO_4_ (1 mg ml^−1^, final concentration 150 μM) and BTTAA (10 mg ml^−1^, final concentration 300 μM) are first mixed and added to the fluorescent probe (TAMRA-Alk or TAMRA-N_3_, 1 mg ml^−1^, desired final concentration). K_2_HPO_4_ (100 mg ml^−1^, final concentration 100 mM) and H_2_O are then added. Finally, sodium ascorbate (10 mg ml^−1^, final concentration 2.5 mM) is added right before use.

### Microplate fluorescence

Overnight cultures were adjusted to OD_600_ 1 by diluting the suspensions with PBS. 200 μl of these suspensions were split in 2 ml microtubes (minimum of 3 per condition) then centrifuged (2 min, 10 000 G), and pellets were resuspended in 200 μl CuAAC buffer and agitated for 45 min, 600 rpm at room temperature in the dark. These suspensions were rinsed 3 times with 1 ml PBS (2 min, 10 000 rpm) before being resuspended in 200 μl PBS. CuAAC whole cell suspensions were split in a dark opaque 96-well plate (100 μl per well). Fluorescence (*λ*_em_ 535 ± 20 nm/*λ*_exc_ 585 ± 30 nm) was measured on a CLARIOstar Plus microplate reader.

### Fluorescence microscopy

Agar pads were made by pouring 10 μl of hot LB agar on a microscopy slide and quickly covering it with a glass coverslip. Whole-cell suspensions were deposited on the hardened agar pad by gently raising the coverslip and placing it back down. These slides were then observed on a Leica AF6000 LX inverted video microscope with differential interference contrast (DIC). For immunofluorescence staining of the capsule, overnight cultures were adjusted to 1 OD_600nm_ by diluting the suspensions with PBS and treated with anti-K1 rabbit antibody (ENZ-ABS559-0100 Enzo Life Sciences) (1/100, 45 min), rinsed with 200 μl PBS and treated with anti-rabbit AF488 antibody (1/250, 30 min), rinsed with 200 μl PBS and finally mounted on agar pads and observed as described above. For CuAAC fluorescence labelling, cells were treated as described for the microplate fluorescence with the fluorophore concentration adjusted to 100 mM. After the reaction and rinsing steps, cells were resuspended in 200 μl PBS and 10 μl of these suspensions were mounted on agar pad before observation on a fluorescence videomicroscope.

### Viability assay and growth curves

For viability assay, OD_600_ of overnight cultures was harmonized to 1 by diluting the suspensions with sterile PBS. 200 μl of these suspensions were transferred in a 96-well plate. Serial dilutions ranging from 10^−1^ to 10^−10^ were performed. 20 μl of these suspensions were streaked on LB agar Petri dishes and grown overnight at 37 °C. Petri dishes showing between 5 to 250 colonies were selected, colonies counted, and the number of CFU in the original suspension was deducted from the dilution. For growth curves, from an isolated colony, 2 ml of LB liquid culture were grown in a 15 ml conical tube for 2 hours at 37 °C. 10 μl of this suspension was used to inoculate 10 ml LB supplemented with the desired chemical reporter or monosaccharide in a 50 ml conical tube. OD_600_ of these suspensions was then recorded at regular intervals.

### Capsule extraction and quantification

Capsular extracts were purified by following a method described previously.^37^ Overnight grown cultures were suspended in 600 μl lysis buffer (100 mM SDS, 50 mM Tris, 0.128 mM NaCl). 600 μl phenol/chloroform/isoamyl alcohol (25 : 24 : 1) were added and agitated 15 min at 65 °C. After centrifugation (16 000 g for 15 min at 4 °C), the upper phase was transferred and completed with 600 μl of ice-cold absolute ethanol. The tubes were stored overnight at −20 °C. Tubes were then centrifuged (10 min, 13 000 g) and the white precipitate was rinsed with ethanol then dried under N_2_ flow. 20 μl DNAse was added (45 min, 37 °C, 300 rpm) followed by 20 μl Proteinase K (1 h, 56 °C, 300 rpm). 560 μl H_2_O and 600 μl phenol/chloroform/isoamyl alcohol (25 : 24:1) were added. Tubes were centrifuged (15 min, 13 000 rpm) and the upper phase transferred in a new microtube. 200 μl H_2_O, 50 μl sodium acetate 3 M and 1 m absolute ethanol were added and the tubes were stored at −20 °C overnight. The tubes were centrifuged (15 min, 13 000 rpm) and the white precipitate was rinsed with 1 ml ice cold absolute ethanol and dried under N_2_ flow. Capsular extracts were finally resuspended in 50 μl of H_2_O. The capsule extracted was labelled and quantified on a microplate reader as follow: 10 μl of the capsular extract solutions were added to 190 μl CuAAC buffer in a microtube and agitated 45 min, 600 G at RT. After the reaction 50 μl sodium acetate 3 M and 1 ml ice cold absolute ethanol were added and tubes were stored overnight at −20 °C. Tubes were then centrifuged (15 min, 13 000 G and the white pellet was rinsed with ice cold absolute ethanol, dried under N_2_ flow, resuspended in 20 μl H_2_O and transferred to a dark opaque 96-well plate for fluorescence readout on a microplate reader. The purity of the capsular extract was performed by polyacrylamide gel electrophoresis, for this 10 μl of the same capsular preparation were loaded on a 15% TBE-PAGE (Tris-Boric-EDTA polyacrylamide gel electrophoresis) as described previously.^51^ The capsular polysaccharides were stained with 5% alcian blue in water for 15 min followed by three washing steps with acetic acid 5% in a solution of 50% methanol until the gel background get unstained.

## Conclusions

We developed a method for the specific bioorthogonal labelling of K1 capsules in *E. coli* after the metabolic incorporation of ManNAc analogues equipped with alkyne and azide chemical handles. These sialylation reporters did not exert any significant effect on bacterial growth. ManNAz was determined as the better choice among the tested reporters, as ManNAl showed inherent long-term cytotoxicity. While both ManNAc and Neu5Ac derivatives were readily incorporated in growing *E. coli* EV36, resulting in fluorescent labelling of whole bacteria, only the signal obtained from ManNAc chemical reporters could be attributed to their incorporation into the polysialic acid capsule. This allowed us to refine our understanding of the capsule metabolic pathways. The method was miniaturized as a microplate assay amenable to screening approaches. With the ability to track the K1 capsule biosynthesis, this platform might be a useful tool for future studies aiming at impacting capsule expression, which is of great interest in the context of increasing pathogen resistance.

## Author contributions

V. R. and Y. R. performed the experiments. V. R., Y. R. and C. L. analysed and interpreted the data. C. L., Y. R., and V. R. wrote the manuscript. V. R. and C. L. prepared figures. C. L. and C. B. conceptualised the project. C. B. and C. L. acquired funding and supervised the work. C. L., V. R. and Y. R. revised the paper.

## Conflicts of interest

There are no conflicts to declare.

## Supplementary Material

CB-004-D2CB00219A-s001
