# Supplementary material for: A bioorthogonal chemistry approach to detect the K1 polysialic acid capsule in Escherichia coli
Source: RSC Chem Biol. 2022 Dec 22;4(2):173–83. doi: 10.1039/d2cb00219a (PMC9906323; doi:10.1039/d2cb00219a)
Supplement: CB-004-D2CB00219A-s001 [file CB-004-D2CB00219A-s001.pdf]

Electronic Supplementary Material (ESI) for RSC Chemical Biology.
This journal is © The Royal Society of Chemistry 2022

Supporting Information

A bioorthogonal chemistry approach to detect the K1 polysialic acid capsule in *Escherichia coli*†

Vincent Rigolot,‡ Yannick Rossez,‡* Christophe Biot,* Cédric Lion*

Univ. Lille, CNRS, UMR 8576 - UGSF - Unité de Glycobiologie Structurale et Fonctionnelle, Lille, France. Email: cedric.lion@univ-lille.fr, christophe.biot@univ-lille.fr and yannick.rossez@univ-lille.fr

† Electronic Supplementary Information (ESI) available. See DOI: 10.1039/x0xx00000x

‡ These authors contributed equally to this work.

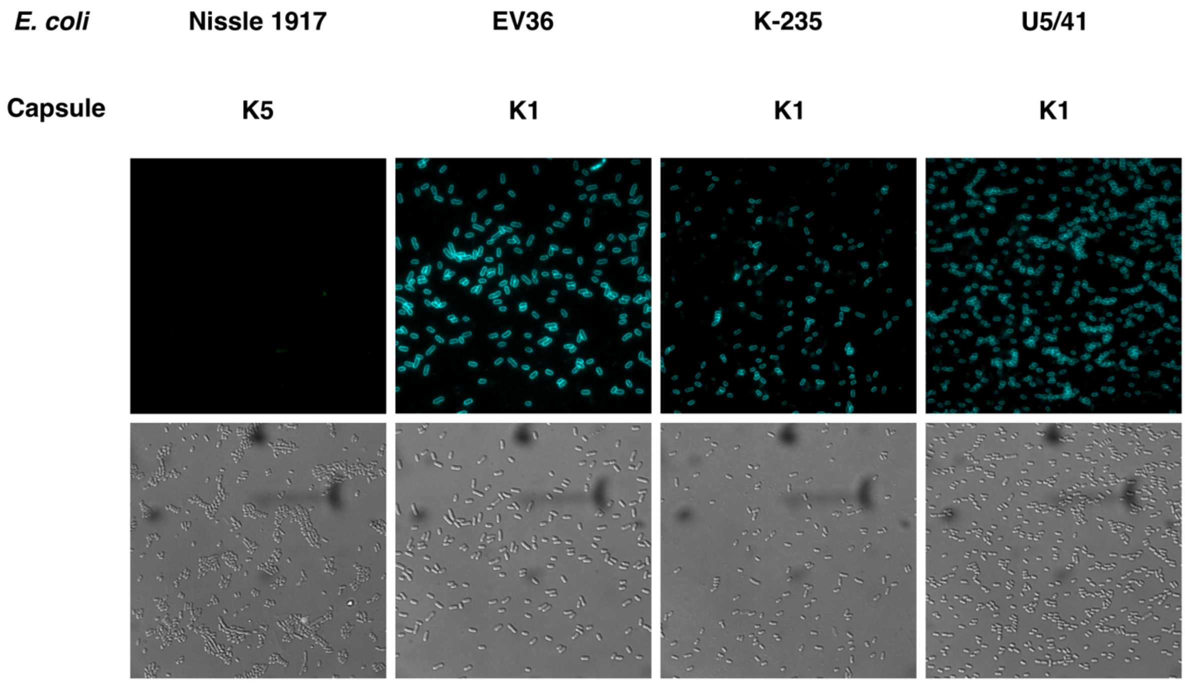

Figure S1. Immunofluorescence microscopy assay. *E. coli* strains Nissle 1917 expressing the K5 capsular polysaccharide (heparosan), and EV36, K-235 and U5/41 expressing the K1 capsular polysaccharide (PSA), grown with ManNAz at 600 µM overnight. Cells were then labelled with a primary anti-K1 antibody (rabbit) then an Alexa Fluor 488 conjugated secondary antibody (anti-rabbit). (top) fluorescence channel showing peripheral staining of K1 expressing cells while the K5 expressing cell is not stained ; (bottom) Brightfield channel.
